# Motion Compensation for 3D Multispectral Handheld Photoacoustic Imaging

**DOI:** 10.3390/bios12121092

**Published:** 2022-11-29

**Authors:** Chiho Yoon, Changyeop Lee, Keecheol Shin, Chulhong Kim

**Affiliations:** 1 Department of Electrical Engineering, Pohang University of Science and Technology (POSTECH), Pohang 37673, Republic of Korea; 2 Department of Mechanical Engineering, Pohang University of Science and Technology (POSTECH), Pohang 37673, Republic of Korea; 3 LIG Nex1, Yongin-si 13488, Republic of Korea; 4 Departments of Electrical Engineering, Convergence IT Engineering, and Mechanical Engineering, Medical Science and Engineering, Pohang University of Science and Technology (POSTECH), Pohang 37673, Republic of Korea

**Keywords:** motion compensation, spectral unmixing, multi-wavelength imaging, photoacoustic, ultrasound

## Abstract

Three-dimensional (3D) handheld photoacoustic (PA) and ultrasound (US) imaging performed using mechanical scanning are more useful than conventional 2D PA/US imaging for obtaining local volumetric information and reducing operator dependence. In particular, 3D multispectral PA imaging can capture vital functional information, such as hemoglobin concentrations and hemoglobin oxygen saturation (sO_2_), of epidermal, hemorrhagic, ischemic, and cancerous diseases. However, the accuracy of PA morphology and physiological parameters is hampered by motion artifacts during image acquisition. The aim of this paper is to apply appropriate correction to remove the effect of such motion artifacts. We propose a new motion compensation method that corrects PA images in both axial and lateral directions based on structural US information. 3D PA/US imaging experiments are performed on a tissue-mimicking phantom and a human wrist to verify the effects of the proposed motion compensation mechanism and the consequent spectral unmixing results. The structural motions and sO_2_ values are confirmed to be successfully corrected by comparing the motion-compensated images with the original images. The proposed method is expected to be useful in various clinical PA imaging applications (e.g., breast cancer, thyroid cancer, and carotid artery disease) that are susceptible to motion contamination during multispectral PA image analysis.

## 1. Introduction

Photoacoustic (PA) images are reconstructed using ultrasound (US) signals generated by localized thermal expansion and contraction induced by the irradiation of light-absorbing imaging targets using a pulsed laser. As intrinsic optical absorbers present in human tissues, such as oxy-hemoglobin (HbO_2_), deoxy-hemoglobin (Hb), lipids, melanin, and water, exhibit unique light absorption coefficients depending on the laser wavelength, PA imaging can emphasize the spectroscopic analysis of biological tissues [1,2,3,4,5,6,7,8,9,10,11,12,13,14,15]. In addition, a spectral unmixing process using multi-wavelength PA images enables the extraction of concentrations of specific light absorbers and captures consequent functional information, such as total hemoglobin concentration, hemoglobin oxygen saturation (sO_2_), and lipid concentration [16,17,18,19,20,21,22,23,24,25,26,27,28]. Thus, multispectral PA imaging can aid the diagnosis of various clinical conditions, such as epidermal, hemorrhagic, ischemic, cancerous, and peripheral diseases [29,30,31,32,33,34,35]. Multi-wavelength PA imaging can be implemented by combining a pulsed laser system with a commercial US imaging machine and is usually operated in 2D space. However, 3D PA/US imaging systems are preferred because 2D PA/US imaging systems exhibit poor repeatability due to operator dependence. Therefore, in this paper, we present a 3D clinical handheld PA/US imaging scanner managed using a mechanical scanning method. The proposed system is used to acquire 3D PA/US images of various human body parts successfully [36,37]. Several studies have been conducted on quality improvement of PA/US images [38,39,40,41,42,43,44], but the motion artifact problem is a major factor affecting this quality. As 3D images are reconstructed by stacking the beamformed 2D images in the mechanical scan direction, the reconstructed 3D images suffer from motion artifacts owing to the breathing and shaking of the image object. Motion artifacts contaminate not only the structural PA image, but also the functional information. Errors induced in physiological information, such as sO_2_, are more severe because the current spectral unmixing process is based on a per-pixel approach, based on the following equation:

(1) $$P=\left[\begin{array}{c} p\left(\lambda_{1}\right)\\ p\left(\lambda_{2}\right)\\ \vdots \\ p\left(\lambda_{N}\right) \end{array}\right]=\left[\begin{array}{cc} \epsilon_{\mathrm{ab}1}\left(\lambda_{1}\right) & \epsilon_{\mathrm{ab}2}\left(\lambda_{1}\right)\\ \epsilon_{\mathrm{ab}1}\left(\lambda_{2}\right) & \epsilon_{\mathrm{ab}2}\left(\lambda_{2}\right)\\ \vdots & \vdots \\ \epsilon_{\mathrm{ab}1}\left(\lambda_{N}\right) & \epsilon_{\mathrm{ab}2}\left(\lambda_{N}\right) \end{array}\cdots \begin{array}{c} \epsilon_{\mathrm{abQ}}\left(\lambda_{1}\right)\\ \epsilon_{\mathrm{abQ}}\left(\lambda_{2}\right)\\ \vdots \\ \epsilon_{\mathrm{abQ}}\left(\lambda_{N}\right) \end{array}\right]\left[\begin{array}{c} C_{\mathrm{ab}1}\\ C_{\mathrm{ab}2}\\ \vdots \\ C_{\mathrm{ab}Q} \end{array}\right]=\mathrm{MC},$$

where N denotes the number of wavelengths used, Q denotes the number of absorbers to be unmixed, p(λ_i_) denotes the PA amplitude at wavelength λ_i_, $\epsilon_{\mathrm{abj}}(\lambda_{i}$) denotes the molar extinction coefficient of absorber j at wavelength $\lambda_{i}$, and $C_{\mathrm{abj}}$ denotes the relative concentration of absorber j. The concentrations are estimated using the pseudo-inverse of M on both sides of the equation. By Equation (1), the concentrations of absorbers at a single pixel location are identical in the multi-wavelength image being scanned. Motion contamination degrades the accuracy of spectral unmixing by disturbing the constancy of concentrations of absorbers at a single pixel location in the scanned multi-wavelength image. To resolve this problem, various motion compensation methods have been proposed. Erlöv et al. performed in vivo PA/US imaging and compensated for motion contamination in PA images using a phase-tracking algorithm on interleaved 2D US images. However, the authors did not demonstrate a reliable reference in US images for motion compensation, and their performance evaluation was limited to single-wavelength PA analysis [45]. Mozaffarzadeh et al. conducted various single-wavelength PA/US imaging experiments on phantoms, both ex vivo and in vivo, and motion-corrected the PA/US images using the modality independent neighbourhood Descriptor (MIND) algorithm. However, as the MIND algorithm performs compensation based on self-similarity, the effect of motion compensation is significantly reduced when motion pollution is severe [46]. Lee et al. presented motion compensation using a low-pass filter to correct spike signals induced by respiration. They reported motion-compensated spectral unmixing results in vivo, but did not present quantified results, and the motion compensation was axially limited [47]. Kirchner et al. reported motion-compensated spectral unmixing in vivo using quantification. Compensation of the PA images was implemented using optical flow, and the flow information was obtained by measuring the brightness patterns in the US images. However, evaluation of spectral unmixing was limited to a 2D imaging system, and the authors compensated for the motion based solely on brightness values [48].

In this study, we present a novel motion compensation method for 3D scanned PA images in both axial and lateral directions. Motion compensation is applied sequentially to all B-mode US images to maximize the structural similarity between successive pairs of B-mode US scan images in terms of the structural similarity index measure (SSIM). SSIM measures and compares the luminance, contrast, and structure of images to quantify similarity [49,50]. Epidermal profiles acquired from US images are used as references for motion correction along the axial direction, whereas US images obtained using the MIND correction algorithm are used as references for lateral motion compensation. During each motion compensation step, the PA motion artifacts are corrected using US motion compensation information. The motion compensation performances in both phantom and in vivo human imaging experiments are evaluated. Comparing the original images with the motion-compensated ones confirms that the PA structure and sO_2_ images are successfully calibrated even in the presence of severe motion contamination. Based on these results, the proposed motion compensation method is expected to be useful for 3D multispectral PA/US imaging of various clinical diseases that are particularly vulnerable to motion contamination owing to tremors induced by breathing or unstable patient conditions.

## 2. Materials and Methods

### 2.1. 3D Clinical Handheld PA/US Imaging System and Scanner

**Figure 1:**
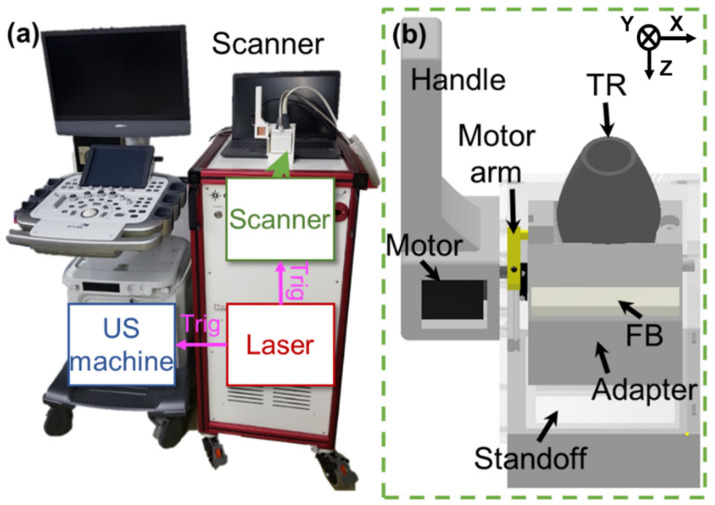
(**a**) Photograph of a clinical handheld PA/US imaging system and a 3D handheld imaging scanner. (**b**) Schematics of the 3D handheld scanner. PA: photoacoustic; US: ultrasound; TR: ultrasound transducer; and FB: fiber bundles.

Figure 1a depicts a photograph of the clinical PA/US imaging system and a 3D handheld imaging scanner. The clinical imaging system consists of a US machine (EC-12R, Alpinion Medical Systems, Seoul, Republic of Korea) and a tunable pulsed laser system (Phocus Mobile, OPOTEK, Carlsbad, CA, USA). The schematic diagram of the 3D handheld scanner is depicted in Figure 1b. The scanner comprises the handle, motor arm, motor (PKP523N12A, Ina Oriental Motor, Tokyo, Japan), adapter, standoff, US transducer (TR) (L3-12, Alpinion Medical Systems, Gangseo-gu, Republic of Korea), and fiber bundles (TFO-VIS100SL46-2000-F, TAIHAN FIBEROPTICS, Gyeonggi-do, Republic of Korea). The US machine has 64 receiving channels and the US transducer has 128-element linear array. Because the number of receiving channels is one-half of the number of US transducer elements, one B-mode PA/US image is obtained with two laser shots. The tunable pulsed laser system uses a pulse repetition frequency (PRF) of 10 Hz, so the final image acquisition speed is 5 frames per second. The fast tuning function of the tunable pulse laser system can tune the wavelength every pulse, and 3 wavelengths are used for spectral unmixing of multi-wavelength PA images. In addition, to safely obtain a single PA image within the transducer’s elevational resolution, the scanning step size ranges between 0.06 mm and 0.11 mm, much smaller than the elevational beam-width of the linear array US transducer (approximately 1 mm at a depth of 30 mm). 3D PA/US imaging and scanning are performed simultaneously when the laser delivers simultaneous triggers to the US machine and scanning system. The US machine displays B-mode PA/PA maximum amplitude projection (MAP) images or B-mode US/US MAP images in real time and simultaneously saves PA radiofrequency (RF)/US image data during the implementation of 3D imaging. Detailed specifications of the equipment, scanning, and data acquisition procedures are presented in [36].

### 2.2. 3D Clinical Handheld PA/US Imaging System and Scanner

**Figure 2:**
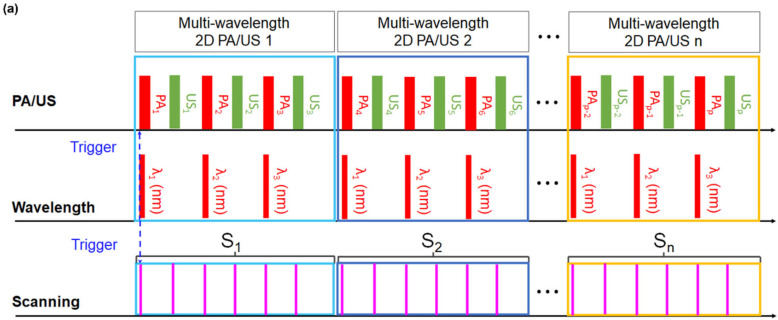

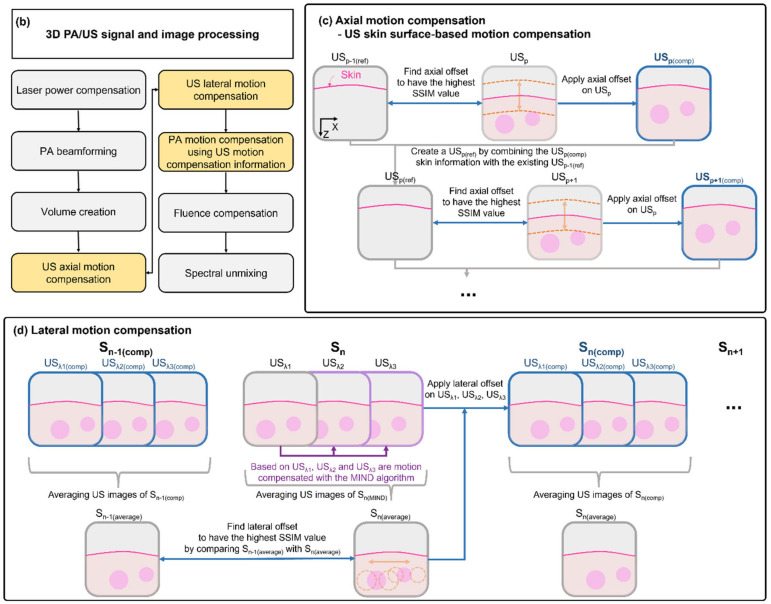
Schematics of (**a**) acquisition of 3D multi-wavelength PA/US images, (**b**) 3D PA/US signal and image processing, (**c**) axial motion compensation, and (**d**) lateral motion compensation. US_p_, US_p(ref)_, and US_p(comp)_ represent the pth acquired US image, the reference image for the following axial motion compensation of US image, and the axial-motion-compensated US image, respectively. S_n_, S_n(MIND)_, S_n(average)_, and S_n(comp)_ represent the US images included in the nth scanning period, the MIND-corrected US images of the nth scanning period, the average US image of the nth scanning period, and the lateral-motion-compensated US images of the n^th^ scanning period, respectively. US_λ1_, US_λ2_, and US_λ3_ represent the US images obtained at three optical wavelengths (756, 797, and 866 nm, respectively). Note that the US images are not affected by the optical wavelengths. PA: photoacoustic; US: ultrasound; ROI: region of interest; SSIM: the structural similarity index measure; and MIND: modality independent neighbourhood descriptor.

The acquisition of 3D multiwavelength PA/US images is illustrated in Figure 2a. When trigger signals are fired from the laser system to the mechanical scanning system and US imaging system simultaneously, 3D imaging and scanning are performed. During the 3D scanning process, three PA images corresponding to three optical wavelengths (756, 797, and 866 nm) are acquired corresponding to each multispectral data set. Each US image is acquired immediately after capturing the corresponding PA image. After acquiring the 3D PA RF/US image data, 3D signal and image processing are implemented offline, as illustrated in Figure 2b. The PA RF data are compensated based on the laser power measured using the energy meter of the laser system before implementing beamforming (BF). The calibrated PA RF data are reconstructed using a delay-and-sum (DAS) BF [51,52,53,54]. The beamformed B-mode PA and US images are stacked along the elevational direction (i.e., scanning direction) to construct the original PA and US volumes, respectively. During each scanning period (i.e., S_n_ in Figure 2a), three PA images with three optical wavelengths and three US images are acquired. The motion artifact of each US image is axially and laterally corrected, and then the position of the corresponding PA image is compensated based on the motion-corrected US image (Figure 2b). The axial motion is corrected first, followed by the lateral motion. For axial motion compensation, an epidermal surface is detected by using edge detection on the B-mode US image, and the skin surface image is used as the basis for axial motion compensation (Figure 2c). The B-mode US images used for axial motion compensation are individual stacked US images without considering the scanning period (i.e., US_p_ in Figure 2a). As the handheld scanner is firmly pressed onto the skin surface, skin surfaces of all B-mode US images are assumed to be relatively maintained. Using the epidermal image of the previous B-mode US image (US_p-1(ref)_) as a reference image, the epidermal surface of the subsequent B-mode US image (US_p_) is moved axially to identify the axial offset corresponding to the maximum SSIM value between the two images. If the SSIM value is maximized, SSIM value is an indicator for determining the structural similarity of the two images, so it is considered the case with the best correction. Therefore, US_p(comp)_ is acquired by shifting US_p_ in the axial direction by the axial offset. For subsequent processing, US_p(ref)_ is empirically updated as follows:

(2) $${\mathrm{US}}_{p\left(\mathrm{ref}\right)}={\mathrm{US}}_{p-1\left(\mathrm{ref}\right)}\times 0.9+{\mathrm{US}}_{p\left(\mathrm{comp}\right)\left(\mathrm{skin}\right)}\times 0.1.$$

For the updated US_p(ref)_, only 10% of the total US_p(comp)(skin)_ value is reflected in Equation (2). This is because if the newly corrected information, US_p(comp)(skin)_, has a high reflection rate and unexpected errors, this error could lead a significant impact on the new reference, US_p(ref)_. Further, this can also affect the subsequent motion correction. Thus, instead of being too sensitive to the new reference, US_p(comp)(skin)_, the US_p(ref)_ is generated mainly using the US_p-1(ref)_, reliable data accumulated over multiple motion-corrected frames. This can also prevent continuous reflection of the unexpected errors in the following motion correction process. The updated US_p(ref)_ is used as a new reference for the subsequent process, and this operation is repeated until all B-mode US images are compensated for. Once the axial motion correction is completed, lateral motion compensation is implemented using axially compensated US volume data (Figure 2d). Although the epidermal surface profile is used as a reference for axial motion compensation, no adequate reference is identified for lateral motion compensation. Therefore, an artificial reference is generated using the MIND correction algorithm. Recently, several motion-tracking algorithms for 3D clinical data using various video tracking methods have been proposed [55,56,57]. Among them, the MIND algorithm is the most effective for motion compensation in all directions of images acquired from similar locations [58]. While tuning the three optical wavelengths, three PA images and three US images (i.e., US_λ1,_ US_λ2,_ and US_λ3_) are acquired via simultaneous mechanical scanning. Note that the US images are not affected by optical tuning. Because the scanning step size between the US images ranges between 0.06 mm and 0.11 mm, which is significantly lower than the elevational beam-width of the US transducer (i.e., ~1 mm), all three US images within a single scanning period (S_n_ in Figure 2a) can be considered to be nearly identical. The MIND correction is one of the image registration methods, which allows both axial and lateral motion correction of images acquired at almost the same location. However, this method is not suitable for the images with large difference. In turns out that the axial motion artifacts are more significant compared to the lateral ones. Therefore, the axial motion is first corrected using the axial motion correction method. Then, the MIND correction is applied for the lateral motion correction to three optical wavelength PA images obtained at the almost same position. The MIND correction can be performed without any reference, such as the epidermal information used in the axial motion correction. Further, the MIND correction allows not only the lateral motion correction but also the additional axial motion correction that may not have been fully corrected in the previous axial motion correction. For the MIND correction, based on the US_λ1_, an offset corresponding to the minimal MIND difference with US_λ2_ is determined, and this offset is applied to obtain the MIND-corrected US_λ2_ (US_λ2(MIND)_). This process is repeated to acquire MIND-corrected US_λ3_ (US_λ3(MIND)_). Using this MIND correction, the images in one scanning period have completed the lateral motion correction, but the lateral motion between the scanning periods has not been corrected. Therefore, all MIND-corrected US images in one scanning period are averaged to generate an artificial reference for additional lateral motion compensation between scanning periods:

(3) $$S_{n\left(\mathrm{average}\right)}=\left({\mathrm{US}}_{\lambda 1\left(\mathrm{MIND}\right)}+{\mathrm{US}}_{\lambda 2\left(\mathrm{MIND}\right)}+{\mathrm{US}}_{\lambda 3\left(\mathrm{MIND}\right)}\right)/3,$$

Based on the previous S_n-1(average)_, each subsequent S_n(average)_ is moved laterally to determine a lateral offset corresponding to the maximum SSIM value. This lateral offset is applied to each MIND-corrected US_λ1(MIND)_, US_λ2(MIND)_, and US_λ3(MIND)_ to obtain laterally motion-corrected US_λ1(comp)_, US_λ2(comp)_, and US_λ3(comp)_ images, respectively. This processing is repeated until all US images are laterally motion-compensated. The motion-compensated PA volume is obtained by applying the motion-compensated US volume. For fluence compensation, the signal and background regions of interest (ROIs) are manually segmented at equal depths. The segmented signal area of each B-mode PA is compensated using the average value of the segmented background area. To obtain PA sO_2_ in the signal area, nonnegative spectral unmixing is performed using fluence-compensated PA signals at each wavelength [41,59]. Oxy-hemoglobin (HbO) and deoxy-hemoglobin (Hb) can be obtained using spectral unmixing, and sO_2_ is the ratio of HbO to total hemoglobin, HbT (sum of HbO and Hb).

## 3. Results and Discussion

### 3.1. Performance Test in Phantoms

**Figure 3:**
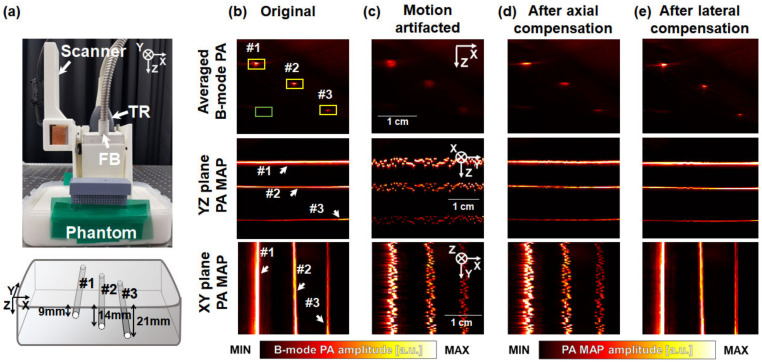

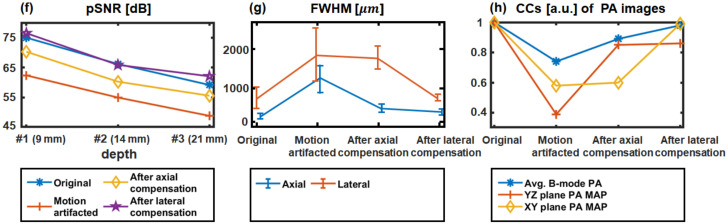
(**a**) Photograph of the 3D handheld scanner placed on the phantom and the phantom schematics. #1-#3 represent three black threads at different depths of 9, 14, and 21 mm, respectively. Yellow and green boxes represent signal and noise areas, respectively. (**b**) Original and (**c**) artificially motion-disrupted photoacoustic (PA) images: the average B-mode PA images along the Y axis, and the PA maximum amplitude projection (MAP) images along the YZ and XY planes, respectively. The average B-mode PA images and PA MAP images along the YZ and XY planes after (**d**) axial and (**e**) lateral motion correction. The X, Y, and Z directions represent lateral, elevational, and axial directions, respectively. The boxes are selected to calculate peak signal-to-noise ratios (pSNRs). (**f**) The pSNRs of the black threads at the different depths calculated at each step of process. (**g**) The FWHMs of the black thread at 9 mm depth calculated at each step of process. (**h**) The cross-correlations (CCs) between the original PA images and others. The original images are used as references to calculate CCs. TR: ultrasound transducer; FB: fiber bundles.

To evaluate the performance of the proposed motion-correction technique, we image a phantom containing three 90-μm black threads at different depths (Figure 3a). The details of the phantom components and the resultant effective attenuation coefficient are explained in [36]. The 3D handheld scanner is placed on the phantom and scanning is performed along the elevational direction (Y-direction). A single laser wavelength of 797 nm with a pulse energy of 8.8 mJ/cm^2^ is used, which does not exceed than the American National Standards Institute (ANSI) safety limit of 31.3 mJ/cm^2^ corresponding to the wavelength. The scanning range, field of view (FOV), and scanning time are taken to be 25.0 mm, 25.0 × 38.4 mm^2^, and 16.7 sec, respectively. Initially, the phantom is imaged statically to obtain the original 3D PA/US images (Figure 3b), and then the phantom is artificially disrupted during scanning to obtain the 3D PA/US images containing motion artifacts (Figure 3c). In the artificial motion disruption process, a motion was applied to the images of all frames in consideration of the actual scanning period during which the image is acquired. In addition, since the motion in the actual situation may have motion not only in the axial direction but also in the lateral direction, we give artificial motion in both axial and lateral directions. During image processing, the axial motion artifacts are corrected first (Figure 3d), followed by the lateral motion artifacts (Figure 3e). All motion correction algorithms are initially performed on US images (data not shown) and subsequently applied to PA images. The performances of the processes are quantitatively compared by quantifying the peak signal-to-noise ratio (pSNR) and full width at half maximum (FWHM) in the axial (i.e., the Z direction) and lateral (i.e., the X direction) directions. The yellow and green boxes in the figure represent the signal and noise regions, respectively, where the pSNRs are calculated. In addition, cross-correlations (CCs) are measured to evaluate the structural similarity between the original images and others. Three types of PA images are depicted in Figure 3b–e: the averaged B-mode PA images along the X-axis and PA maximum amplitude projection (MAP) images on the YZ and XY planes. The thread images are clearly visualized in the original images (Figure 3b), whereas the structures are disturbed in the images containing motion artifacts in all directions (Figure 3c). After axial motion correction, the thread structures are clearly axially, but not laterally, corrected (Figure 3d). After lateral motion correction, the remaining lateral artifacts are precisely corrected (Figure 3e). 

The pSNRs quantified from the three black threads for each image-processing procedure are depicted in Figure 3f. The averaged pSNRs of the image processing methods are 66.8 ± 7.9, 55.4 ± 6.7, 62.1 ± 7.5, and 68.2 ± 7.4 dB, respectively. As the threads are originally arranged as straight lines along the scanning direction, their positions in each image along the scanning direction coincide progressively with motion compensation, resulting in a progressive improvement in pSNR. The axial and lateral FWHMs of the thread at 9mm depth are depicted in Figure 3g. To quantify the motion correction in the scanning direction with FWHM, all frames are cut into nine intervals to obtain nine averaged B-mode PA images. Then, calculate the axial and lateral FWHMs of the nine averaged B-mode PA images. The FWHM results in Figure 3g are the mean and standard derivation of these FWHM values. The axial profiles are 241 ± 9, 1239 ± 380, 315 ± 31, and 258 ± 16 μm, respectively, in image processing order. The axial motion correction method maximally corrects artifacts along the axial direction. Interestingly, axial correction is further improved via the lateral correction process because the MIND correction during lateral correction additionally improves the axial profile. The lateral profiles are 790 ± 374, 1845 ± 689, 1811 ± 259, and 704 ± 64 μm, respectively, in image processing order, where the maximal improvement in the latter profile is observed after lateral motion correction. The CCs between the original and other PA images are presented in Figure 3h, revealing that the structural similarity gradually improves as motion compensation proceeds and becomes almost equal to 1 after the completion of motion correction.

### 3.2. In Vivo 3D Multi-Wavelength PA/US Imaging of a Human Wrist

**Figure 4:**
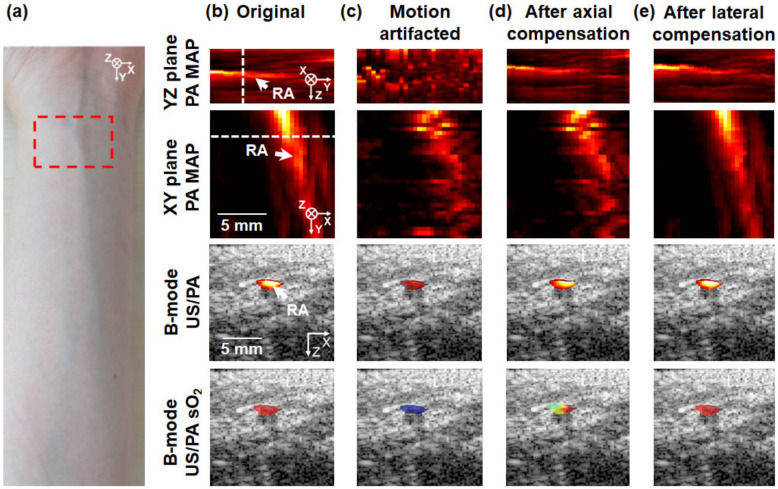

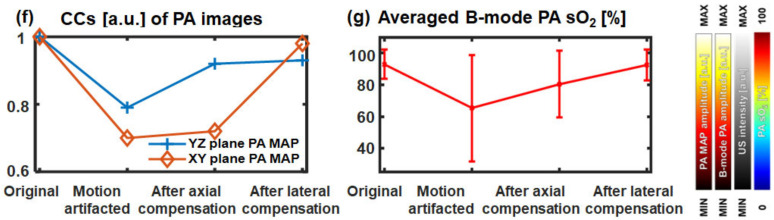
In vivo 3D multispectral PA/US imaging of a human wrist. (**a**) Photograph of the human wrist. The red dashed boxes represent the imaged regions. (**b**) Original and (**c**) artificially motion-disrupted photoacoustic (PA) maximum amplitude projection (MAP) images on the YZ and XY planes of the human wrist. The PA MAP images on the YZ and XY planes after (**d**) axial and (**e**) lateral motion correction. The X, Y, and Z directions represent lateral, elevational, and axial directions, respectively. B-mode US/PA and B-mode US/PA sO_2_ images of the human wrist are presented. The PA and PA sO_2_ images of the upper boundaries of radial arteries (RAs) are overlaid on the corresponding US images. White dashed lines in the MAP images correspond to B-mode US/PA and B-mode US/PA sO_2_ image positions. (**f**) The cross-correlations (CCs) between the original PA images and others. (**g**) The average PA sO_2_ values in the RAs. sO_2_: haemoglobin oxygen saturation.

To investigate the practical applicability and performance of the proposed motion correction methodology, a human wrist is imaged (Figure 4). 3D PA/US data and spectral unmixing results of the radial vessels are obtained by performing 3D multispectral PA/US imaging using a 3D handheld scanner (Figure 4a). The experiments are performed following the protocols of the Institutional Review Board of the Pohang University of Science and Technology (POSTECH, PIRB-2020-E019). 756, 797, and 866 nm laser wavelengths are used for multiwavelength PA imaging—756 and 866 nm are selected based on 797 nm, the isosbestic points of oxy- and deoxy-hemoglobin, respectively. The pulse energy of the 756 nm wavelength providing maximum pulse energy, 10.7 mJ/cm^2^, which does not exceed the ANSI safety limit of 25.9 mJ/cm^2^ [37]. Safety goggles are worn by the volunteer and examiner to prevent exposure to the laser. The scanning range and FOV are identical to those in the phantom experiments, and the scanning time is three times greater than that in the phantom experiments. Radial arteries (RAs) and radial veins (RVs) are primarily used for sO_2_ quantification. The PA MAP images are segmented into 5.8–12.2 mm and 3.9–13.2 mm segments in the axial and lateral directions, respectively, at positions with clearly visible RAs in the PA MAP images. The segmented images are visualized in Figure 4. Previous studies have reported that arterial and venous sO_2_ values increase and decrease after motion compensation, respectively, compared to those prior to motion compensation [48]. To analyze the spectral unmixing effect on the ROI without confusion, the imaging sections where the RA and RV are not clearly distinguished are excluded. The upper boundaries of the RA in each B-mode PA image are manually segmented using MATLAB (MATLAB R2021b, MathWorks, Portola Valley, CA, USA), with reference to the corresponding B-mode US images. To implement fluence compensation, the normal tissue regions are manually segmented at the same depth as the RA of each B-mode PA image. For each B-mode image, the average normal tissue value is used to compensate for the PA signals in the RA [59]. The B-mode PA sO_2_ values in RA are calculated using spectral unmixing with fluence-compensated PA signals. The top 50% of sO_2_ values present in the PA RA signals are averaged to obtain a representative sO_2_ value. The PA images acquired at 866 nm are used as representative PA images.

First, the human wrist is statically imaged to obtain the original 3D PA/US images (Figure 4b), and then it is artificially dislocated during scanning to obtain the 3D PA/US images containing motion artifacts (Figure 4c). To achieve motion compensation, the axial motion artifacts are first corrected, followed by the lateral artifacts (Figure 4d,e). As in the case of the phantom, all motion corrections are initially performed on US images and then applied to PA images. Four types of images are identified in each image-processing step, as depicted in Figure 4b–e: the PA MAP images on the YZ and XY planes, the B-mode US/PA image, and the US/PA sO_2_ image. The PA MAP images of the YZ and XY planes reveal gradual improvements in structure in terms of the image quality over the image processing steps. The PA MAP image of the YZ plane reveals that the RA was well corrected in the axial direction after axial correction (Figure 4c), and the PA MAP image of the XY plane reveals that the RA was well corrected in the lateral direction after lateral correction (Figure 4d). In the PA MAP images of the YZ and XY planes, the white arrow represents the RA and the white dotted line corresponds to the B-mode US/PA and US/PA sO_2_ image positions (Figure 4b). The B-mode PA structural and PA sO_2_ images of the upper boundaries of the RA are superimposed on B-mode US images. For both the B-mode US/PA and US/PA sO_2_ images, the images are observed to improve gradually, similar to the original image, as the motion correction progresses. In particular, in the case of the US/PA sO_2_ image (Figure 4d) containing motion artifacts, the sO_2_ value is close to zero because of motion artifacts. However, following complete motion correction (Figure 4e), the sO_2_ value becomes nearly equal to the original value, confirming the success of motion correction.

The CCs quantified based on the PA MAP images on the YZ and XY planes obtained via the image-processing procedures are depicted in Figure 4f. The CCs of the PA MAP images on the YZ plane are 1, 0.79, 0.92, and 0.93, respectively, in the order of image processing methods, while those of the PA MAP images on the XY plane are 1, 0.70, 0.72, and 0.98, respectively. These values confirm that the structural similarity eventually improves with progressive motion compensation. The average PA sO_2_ values are 92.9 ± 9.2, 65.3 ± 33.4, 80.4 ± 21.0, and 92.5 ± 9.6%, respectively, in the image processing order (Figure 4g). The average PA sO_2_ values in the RAs also corroborate that the accuracy of spectral unmixing gradually improves with progressive motion compensation.

## 4. Conclusions

In this paper, we propose a new motion compensation method for 3D multispectral PA imaging. Motion compensation is implemented in systematic order using simultaneously acquired US images. The potential of the proposed motion compensation system is confirmed using a tissue-mimicking phantom and in vivo human experiments, both qualitatively and quantitatively. In particular, the accuracy of the spectral unmixing process is improved significantly using the proposed motion correction methodology.

During the acquisition of 3D US/PA images in practical environments, several motion contaminants can occur, induced by factors such as the operator’s breathing and hand tremors, as well as the patient’s breathing and body tremors. Moreover, imaging close to the heart or neck can suffer from motion contamination due to breathing and heartbeat, which can be prevented using an electrocardiogram (ECG) sensor [60]. However, the overall system using ECG becomes more complex, and the influence of other motion pollutants cannot be excluded. Our results indicate that motion compensation using US images can be used in actual clinical environments, where various motion contaminations occur. In future works, we intend to apply motion compensation to 3D multispectral PA images of various diseases such as carotid artery disease, thyroid cancer, and breast cancer in clinical practice.

## Data Availability

The data presented in this study are available on request from the corresponding author.
